# Effectiveness of and Financial Returns to Voluntary Medical Male Circumcision for HIV Prevention in South Africa: An Incremental Cost-Effectiveness Analysis

**DOI:** 10.1371/journal.pmed.1002012

**Published:** 2016-05-03

**Authors:** Markus Haacker, Nicole Fraser-Hurt, Marelize Gorgens

**Affiliations:** 1 World Bank, Washington, District of Columbia, United States of America; 2 Harvard School of Public Health, Harvard University, Boston, Massachusetts, United States of America; Makerere University Medical School, UGANDA

## Abstract

**Background:**

Empirical studies and population-level policy simulations show the importance of voluntary medical male circumcision (VMMC) in generalized epidemics. This paper complements available scenario-based studies (projecting costs and outcomes over some policy period, typically spanning decades) by adopting an incremental approach—analyzing the expected consequences of circumcising one male individual with specific characteristics in a specific year. This approach yields more precise estimates of VMMC’s cost-effectiveness and identifies the outcomes of current investments in VMMC (e.g., within a fiscal budget period) rather than of investments spread over the entire policy period.

**Methods/Findings:**

The model has three components. We adapted the ASSA2008 model, a demographic and epidemiological model of the HIV epidemic in South Africa, to analyze the impact of one VMMC on HIV incidence over time and across the population. A costing module tracked the costs of VMMC and the resulting financial savings owing to reduced HIV incidence over time. Then, we used several financial indicators to assess the cost-effectiveness of and financial return on investments in VMMC. One circumcision of a young man up to age 20 prevents on average over 0.2 HIV infections, but this effect declines steeply with age, e.g., to 0.08 by age 30. Net financial savings from one VMMC at age 20 are estimated at US$617 at a discount rate of 5% and are lower for circumcisions both at younger ages (because the savings occur later and are discounted more) and at older ages (because male circumcision becomes less effective). Investments in male circumcision carry a financial rate of return of up to 14.5% (for circumcisions at age 20). The cost of a male circumcision is refinanced fastest, after 13 y, for circumcisions at ages 20 to 25. Principal limitations of the analysis arise from the long time (decades) over which the effects of VMMC unfold—the results are therefore sensitive to the discount rate applied, and more generally to the future course of the epidemic and of HIV/AIDS-related policies pursued by the government.

**Conclusions:**

VMMC in South Africa is highly effective in reducing both HIV incidence and the financial costs of the HIV response. The return on investment is highest if males are circumcised between ages 20 and 25, but this return on investment declines steeply with age.

## Abstract 2

Medical male circumcision reduces incidences of HIV. Gorgens and colleagues present a new model that identifies the best age for this treatment, in terms of cost efficiencies and disease incidence.

## Editors' Summary

**Background:**

Every year, about 2 million people become infected with HIV, the virus that causes AIDS. Although antiretroviral therapy can keep HIV in check, there is no cure for AIDS. Consequently, prevention of HIV transmission is an important component of efforts to control the AIDS epidemic. Because HIV is most often spread through unprotected sex with an infected partner, individuals can reduce their risk of becoming infected by abstaining from sex, by having only one or a few sexual partners, and by using male or female condoms. In addition, three trials undertaken in sub-Saharan Africa a decade ago showed that male circumcision—the surgical removal of the foreskin, a loose fold of skin that covers the head of the penis—can halve the sexual transmission of HIV from HIV-positive women to HIV-negative men. In 2007, the World Health Organization (WHO) and the Joint United Nations Programme on HIV/AIDS (UNAIDS) recommended that individuals living in countries with generalized HIV epidemics (countries where more than 1% of the general population is HIV positive) should be offered voluntary medical male circumcision (VMMC) to help prevent HIV transmission.

**Why Was This Study Done?:**

In 2011, following several studies that assessed the population-level effectiveness and cost-effectiveness of VMMC scale-up, WHO/UNAIDS set a target of having at least 80% of all men aged 15–49 circumcised in 14 priority countries in Africa by 2015. Good progress towards this target was made, but VMMC scale-up needs further improvement. Scenario-based studies provide estimates of the overall costs and impacts of VMMC policies over the whole policy period, which can span several decades, but policy makers sometimes need to know the returns on investments in VMMC over the current financial year. Here, the researchers use an “incremental” cost-effectiveness approach (one that analyzes the expected consequences of circumcising one male with specific characteristics in a specific year) to provide estimates of the impacts of current VMMC investments and to obtain more precise estimates of VMMC’s cost-effectiveness for HIV prevention in South Africa, a country with a generalized HIV epidemic.

**What Did the Researchers Do and Find?:**

The researchers developed a mathematical model consisting of three elements: a demographic and epidemiological model of the South African HIV epidemic adapted to analyze the impact of one VMMC on HIV incidence over time and across the population; a module to track the costs of VMMC and financial savings from HIV infections averted by VMMC; and several financial indicators to assess the cost-effectiveness of and financial return on investments in VMMC. Using this model, the researchers estimate that the circumcision of a young man up to age 20 prevents, on average, 0.2 HIV infections, and that the number of infections averted by VMMC declines steeply with age at circumcision. Estimated financial savings from one VMMC at age 20 are US$617 at a discount rate of 5% (discounting translates future costs and benefits into present-day values); VMMC at younger and older ages results in lower financial savings. From a purely financial perspective, circumcisions at age 20 are most effective with a financial return of 14.5% (they are equivalent to a financial investment yielding an interest of 14.5% annually). Finally, the costs of a circumcision are refinanced (covered by reductions in the costs associated with HIV infection) fastest for circumcisions at ages 20–25.

**What Do These Findings Mean?:**

Because the effects of VMMC unfold over many decades, the accuracy of these findings is likely to be affected by the future course of the AIDS epidemic, by HIV/AIDS policies adopted by the South African government, and by the discount rate applied in the cost-effectiveness analysis. Moreover, some of the assumptions made by the researchers in their model may affect the accuracy of their findings. Overall, however, these findings, which provide estimates of the consequences of current VMMC policies, suggest that VMMC in South Africa (and possibly in settings where a lower proportion of the population is HIV positive) is a highly effective HIV prevention intervention that is cost-saving under many circumstances. These findings also indicate how age at circumcision affects the impacts of circumcision. Specifically, they suggest that the return on investment is highest if males are circumcised between ages 20 and 25 but declines steeply with increasing age.

**Additional Information:**

This list of resources contains links that can be accessed when viewing the PDF on a device or via the online version of the article at http://dx.doi.org/10.1371/journal.pmed.1002012.

- Information is available from the US National Institute of Allergy and infectious diseases on HIV infection and AIDS
- NAM/aidsmap provides basic information about HIV/AIDS, summaries of recent research findings on HIV care and treatment, information on male circumcision for the prevention of HIV transmission, and personal stories about living with HIV/AIDS
- Information is available from Avert, an international AIDS charity, on many aspects of HIV/AIDS, including information on how HIV is transmitted, on voluntary medical male circumcision for HIV prevention, and on HIV/AIDS in sub-Saharan Africa; Avert also provides personal stories about living with HIV/AIDS
- The World Health Organization provides information on all aspects of HIV/AIDS (in several languages), including information on voluntary medical male circumcision for HIV prevention
- UNAIDS provides up-to-information about the AIDS epidemic and detailed information about HIV/AIDS in South Africa
- The Clearinghouse on Male Circumcision for HIV Prevention provides up-to-date information and resources on male circumcision for HIV prevention

## Introduction

The effectiveness of male circumcision in reducing sexual transmission of HIV from HIV-positive women to HIV-negative men has been generally accepted at least since 2007 [1], after the results of three randomized control trials from Kenya [2], South Africa [3], and Uganda [4] became known. In the same year, the President’s Emergency Plan for AIDS Relief (PEPFAR) started funding voluntary medical male circumcision (VMMC) programs. In 2011, the Joint United Nations Programme on HIV/AIDS (UNAIDS) investment framework [5] recognized male circumcision in generalized epidemics with a low prevalence of male circumcision as one of the “basic program activities” with proven effectiveness in HIV prevention. That year, the World Health Organization (WHO) and UNAIDS launched the “Joint Strategic Action Framework to Accelerate the Scale-Up of Voluntary Medical Male Circumcision” [6], setting a target of reaching a VMMC prevalence of at least 80% among males ages 15–49 in 14 priority countries in eastern and southern Africa by 2015.

These programmatic developments have motivated, and were informed by, a number of studies assessing the population-level effectiveness [7,8], the required costs, and the cost-effectiveness [9–11] of comprehensively scaling up VMMC.

Four general lessons emerge from this academic literature. First, VMMC—in countries with a high burden of HIV and low prevalence of male circumcision—is very effective in averting HIV infections among men. Njeuhmeli et al. [11] estimated that attaining 80% coverage of VMMC across 13 priority countries facing high HIV prevalence by 2015, and maintaining it through 2025, would require 28.8 million circumcisions and avert 3.36 million new HIV infections by 2025. For a similar set of countries, Auvert et al. [10] estimated that each male circumcision would avert 0.25 HIV infections over a 20-y period. Second, not only individuals who become circumcised benefit—the effects are spread across males and females in the population, as circumcised individuals not infected because of VMMC also do not transmit HIV to others [11,12], and so on. Third, the effects of male circumcision grow over time: it is a one-off intervention that reduces the risk of HIV acquisition during every sexual act following circumcision, for life, and any downstream HIV infections among partners and so on also involve some time lag. Fourth, VMMC—at least for the high-prevalence countries the available studies are focusing on—is a cost-saving intervention, as savings in treatment costs owing to HIV infections averted outweigh the costs of VMMC [9–11]. For example, Njeuhmeli et al. [11] estimate that the scale-up of VMMC would cost US$2 billion but would also yield financial savings in treatment costs of US$18.5 billion by 2025, so that the program results in net savings of US$16.5 billion by 2025.

The drive toward expanding coverage of male circumcision has resulted in a steep increase in the number of male circumcisions performed. In 2014, 3.2 million men in the 14 priority countries were circumcised, contributing to a total of 9.1 million circumcisions since 2008 [13]. Nevertheless, the pace of the scale-up of VMMC so far is insufficient to reach a coverage of 80% of males ages 15–49 by 2016 as envisaged in the WHO/UNAIDS Joint Strategic Action Framework [6,14].

The literature on the experience of the scale-up of VMMC so far offers lessons for improving the efficiency of VMMC services, by analyzing cost drivers [15], identifying obstacles to scale-up such as demand-creation challenges [16–18], or addressing access to VMMC for specific age groups (e.g. [19]). The most recent modeling efforts focus on the effects of targeting specific age groups in recognition of the challenges in reaching older males through VMMC programs [20] but also reflecting a shift in the global discourse on HIV prevention toward calibrating HIV prevention efforts more finely to the most important drivers of the HIV epidemic. Some earlier studies differentiated by age at circumcision [8,21]. The most substantial current efforts are the “Decision Makers’ Program Planning Toolkit (DMPPT 2.0)” [22], which is an Excel-based spreadsheet building on inputs from a national (or regional) Spectrum model [23], and the “age-structured model,” which also accounts for sexual risk groups [24].

This paper complements other current efforts to estimate the impacts of male circumcision by age at circumcision but differs in terms of the approach. Rather than assessing the overall costs and impacts of a VMMC policy scenario (specifying the number of circumcisions at different ages performed over some policy period), it estimates the impacts over time and across the population of circumcising one male individual at a specific age in a specific year. The present analysis is related to scenario-based approaches as both rely on a fully specified epidemiological model to generate projections of the state of HIV/AIDS and the effects of the policy contemplated.

However, the purposes and outcomes of the respective analyses differ. Policy scenarios are an effective and intuitive way of describing the costs and consequences of policies under consideration and obtaining some measures of cost-effectiveness (e.g., costs per HIV infection averted over the policy period).

The value added from the incremental analysis arises in two areas, but both are related as they address challenges associated with the long time frames required to capture the effects of investments in VMMC—typically, policy scenarios therefore extend over several decades. In contrast, the incremental approach provides estimates of the impacts of current VMMC investments—e.g., informing policy makers on what the returns are on spending considered as part of the next annual government budget.

Additionally, the incremental analysis offers more precise estimates of the cost-effectiveness of current VMMC policies, for two reasons. First, policy scenarios show average effects of interventions occurring over (possibly long) policy periods and, therefore, do not offer precise estimates of the effects of interventions at any point in time. Second, policy scenarios are subject to a cut-off problem, as some of the effects of interventions studied within the policy period lie outside the policy period. In particular, they barely capture the effects of a VMMC performed late in the period. The incremental analysis gets around these problems by focusing on VMMC policies in a particular period (so there is no averaging over periods), and because it can follow the impacts of one VMMC until it dissipates (so there is no arbitrary cut-off) and capture the full effects.

## Methods

The model used in this analysis consists of three elements: a demographic and epidemiological model used to estimate the projected impacts of one VMMC on HIV incidence, a costing module used to project the costs caused by a new HIV infection (and the savings from HIV infections averted as a consequence of one VMMC), and a financial analysis describing the cost-effectiveness of and the financial returns to investments in VMMC.

### Demographic and Epidemiological Model

The analysis of the effects of VMMC on HIV incidence was conducted using an adapted version of the ASSA2008 national (“lite”) model [25] developed in 2011 and broadly in line with the National Strategic Plan for HIV, TB, and STIs (2012–2016). ASSA2008 is a compartmental HIV disease progression and transmission model of the HIV epidemic in South Africa embedded in a demographic model. For each sex, adults up to age 59 are divided in four risk groups—with risk behaviour similar to sex workers, high-risk behaviour of someone regularly infected with sexually transmitted disease, individuals with a lower level of sexual activity but still at risk, and individuals not at risk (see appendix for more precise definitions), irrespective of ethnicity or geographic location. At younger ages, individuals may move into groups characterized by higher risk; from age 25, the assignment to risk groups is fixed. The population is divided in one-year cohorts, and people living with HIV are also differentiated by time from infection and stage of disease. An alternative version of the ASSA2008 model also allows an analysis by province and main ethnic group (“African,” “Coloured,” “Indian,” and “White”). This expanded model has not been used, because the main comparator analyses are at the national level, the modelling draws from all uncircumcised males in the country, irrespective of their ethnic group or geographic location, and the thrust of the paper is on methodological aspects.

We chose this model, because it allows an incremental analysis such as the one performed here in a relatively straightforward fashion. The ASSA2008 model, however, does not explicitly account for male circumcision and therefore needed to be adapted in four steps (see S1 Text for details): (1) adding some structure to the model to account for the number and age structure of circumcised males (using the model’s existing structure for uncircumcised males); (2) adding assumptions on the historical uptake and prevalence of male circumcision in South Africa, so that the estimates are consistent with current estimates of the uptake of VMMC; (3) adjusting the model to separately account for the risk of infection for circumcised and uncircumcised males; and (4) adjusting the parameters on the risk of female-to-male transmission of HIV, distinguishing the risk for circumcised and uncircumcised males. This was done assuming that HIV acquisition for circumcised males is 60% lower than for uncircumcised males [1] and setting the relevant parameters for uncircumcised males so that the model closely reproduces HIV incidence and prevalence from the unadjusted model. This was achieved by setting the parameters on the risk of HIV acquisition for uncircumcised males at about 1.5 times the parameters of the unadapted model and the corresponding parameters for circumcised males at 40% lower than the original values. The model does not take into account potential impacts of male circumcision on male-to-female HIV transmission, in line with most comparable analyses.

### Cost Estimates

To translate the estimates of the impacts of VMMC on HIV incidence into projections of the resulting changes in the demand for HIV/AIDS services, an epidemiological model describing disease progression and transition into treatment and a costing framework were required. The ASSA2008 model has not been used for this purpose, because it is known to underestimate survival on antiretroviral treatment [26], an important shortcoming for the present purposes. Instead, the disease progression module builds on Spectrum estimates for South Africa and treatment coverage targets from the “National Strategic Plan on HIV, STIs, and TB 2012–2016” [27]. This approach has been similarly used in the costing of the national strategic plan and in another major policy study [28], and allows use of the analytical capabilities of the ASSA2008 model while circumventing known shortcomings. Underestimating survival on treatment could also result in underestimates of HIV transmission in the epidemiological estimates. However, as the ASSA2008 model (similar to the Spectrum model) assumes a large reduction (about 90 percent) in HIV transmission while on treatment, any resulting bias in HIV transmission would be small.

The costs of treatment, which are the dominant aspect of the costs caused by HIV infections, were updated in line with recent estimates produced for the South African HIV/TB investment case [29] and are current as of March 2015, with costs of ZAR 3,101, equivalent to US$267 for first-line treatment, and ZAR 6,155 (US$531) for second-line treatment, applying a year-average exchange rate of ZAR 11.59 per United States dollar as of end-2014. For male VMMC, a unit cost of ZAR 1,210 (US$104) for adults and ZAR 605 (US$52) for infants was assumed, based on recent expenditure data both from the public and private sector used in the South African HIV/TB investment case. Other cost items—such as non-antiretroviral care and disability and foster-care grants—were updated from the earlier estimates [28,30] to 2013 prices, making an adjustment for inflation. Looking forward, all costs except drug costs were assumed to grow at a rate of one percent annually (about the rate of growth of real GDP per capita recorded in South Africa from 2010–2014), to account for rising wages of health workers etc. A markup of 5.5% was applied to all spending categories to account for overhead expenses.

### Financial Analysis

Capturing the effectiveness and cost-effectiveness of VMMC is complicated, because the effects of VMMC on HIV incidence are spread over a long period (decades), and the cost savings from reduced HIV incidence are spread out even further (another several decades). Estimates of the most common indicators (net costs of or savings from one VMMC) are therefore highly sensitive to the discount rate applied, and the main results (applying a discount rate of 5%) are complemented by a sensitivity analysis with regard to the interest rate. Additionally, the analysis offered some indicators more commonly used for the analysis of financial investments—the amortization period (for a given interest rate, how long does it take until the initial costs are refinanced?) and the financial rate of return (up to which interest rate are the financial returns positive, and is the investment in VMMC a good financial investment?).

### Implementation

To estimate the consequences of VMMC occurring in 2013, the number of male circumcisions performed at a specific age in 2013 was raised by one, at age 0 (for early infant male circumcision) and in 5-y increments from age 10 to age 55. The analysis focuses on the consequences of circumcising South African HIV-negative individuals irrespective of ethnicity or geographic location; i.e., it was assumed that the individual circumcised, at each age, was randomly drawn from HIV-negative individuals from the four risk groups at that age. As the HIV status of an individual undergoing circumcision may be unobserved, the analysis was repeated assuming that individuals were drawn randomly from all male individuals at a specific age, regardless of HIV status. The model was then used to project the expected consequences for the individual circumcised and across the population.

The adapted model includes some population-level assumptions on the uptake of male circumcision (see S1 Text). The analysis here assumed that the additional male circumcision performed does not affect the number of male circumcisions at a later date, i.e., the individual undergoing it in the analysis would not otherwise be circumcised later. The direct effect of male circumcision (on the individual circumcised) was calculated by following the cohort for life and calculating the annual HIV infection risk for circumcised and uncircumcised males. The population-level effects were calculated as the difference between the results from the projections, including the additional circumcision, and a baseline not including this perturbation.

## Results

We determined the impact of VMMC on HIV incidence for the individual circumcised and among men and women across the population, identified financial savings accruing from VMMC by age, and incorporated these savings in the analysis of the cost-effectiveness of VMMC, by age of the individual circumcised. The savings are spread over a very long period, with calculations of potential savings sensitive to the discount rate applied. Because of the large magnitude of the financial savings, we also interpreted investments in VMMC as financial investments, applying indicators like the financial rate of return and amortization period.

### Impact of VMMC on HIV Incidence

**Fig 1:**
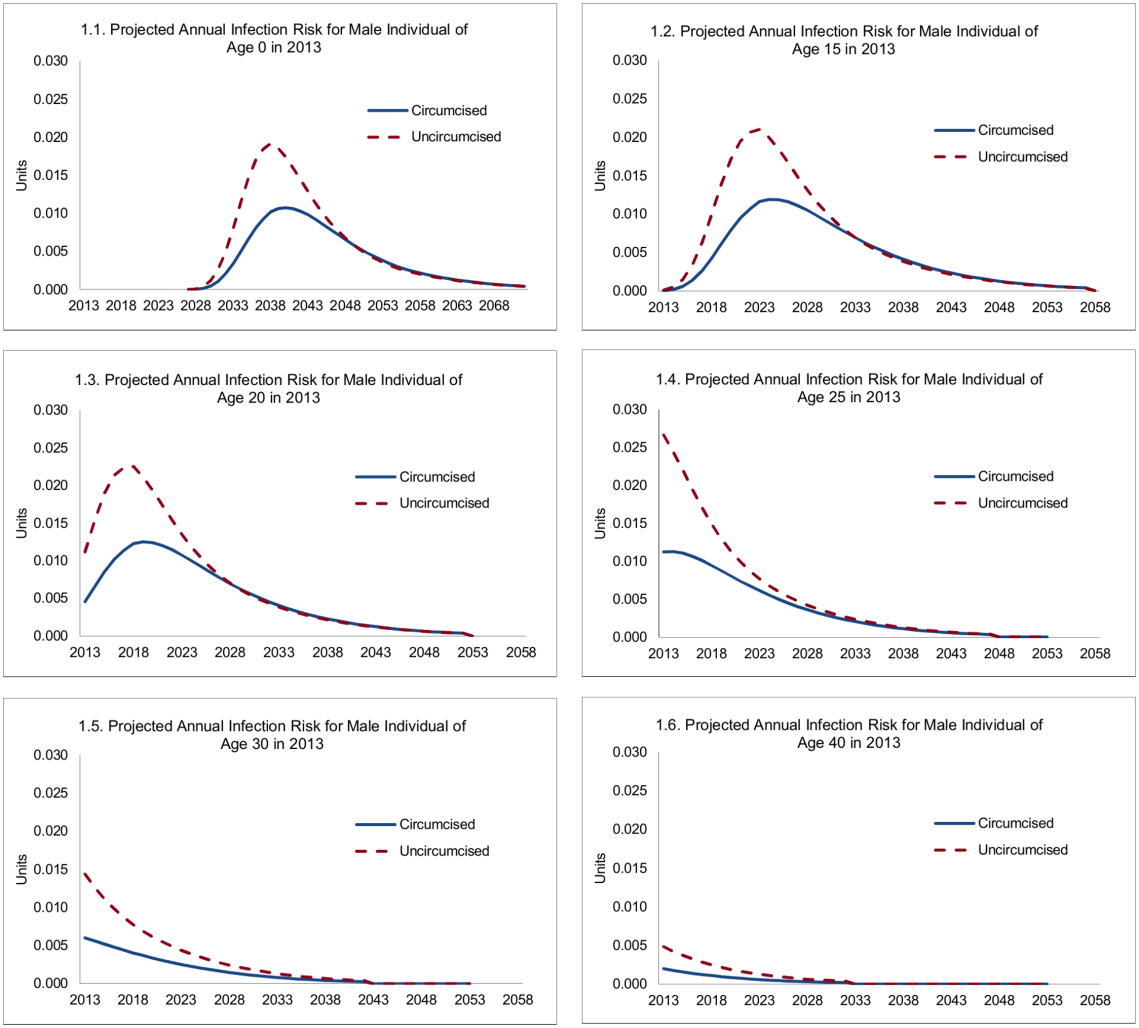
Projected annual infection risk for male individual, by age.

Male circumcision reduces HIV incidence both directly and indirectly—directly, because it reduces the HIV infection risk for the uninfected individual circumcised, and indirectly, because the reduced infection risk for the individual circumcised also reduces the risk that he transmits HIV to sexual partners and so on. The direct effects are summarized in Fig 1, showing the projected infection risk for circumcised and uncircumcised HIV-negative individuals of various ages as of 2013. The projected annual infection risk is shown as contributions to lifetime HIV infection risk. For example, the probability of an HIV-negative, uncircumcised individual who is 20-y-old in 2013 becoming infected in 2020 is 1.9%, which is equal to 2.3% (infection risk in 2020) times 0.83 (the probability of surviving and remaining HIV-negative until 2020). The curves converge in outer years, because lower HIV incidence among circumcised men means that a higher share remains HIV-negative at older ages and may still get infected then. In this presentation, the lifetime risk of contracting HIV is represented by the area underneath the respective graphs in Fig 1, and the reduction in lifetime risk of HIV infection is represented by the area between the graphs for uncircumcised and circumcised individuals.

For an uncircumcised 15-y-old male in 2013, the probability that he will contract HIV in a specific future year steeply increases to 2.1% by age 25, reflecting increasing sexual activity, a shift to higher-risk sexual behavior, and an increasing risk of contact with HIV-positive females. Subsequently, it declines steadily to 1.3% by age 30, 0.4% by age 40, and 0.1% by age 50, reflecting declining sexual activity (for men from age 27) and the changing composition of men not infected with HIV (men reaching older ages without becoming infected are statistically more likely to have adopted, and to adopt, low-risk sexual behavior). For a circumcised male, the probability that he will contract HIV peaks at a much lower rate (1.2%) at age 26. For infant circumcision (male newborns circumcised within a year of birth), the lifetime HIV infection risk is similar to that for a 15-y-old, but occurs with a long delay once individuals become sexually active.

**Fig 2:**
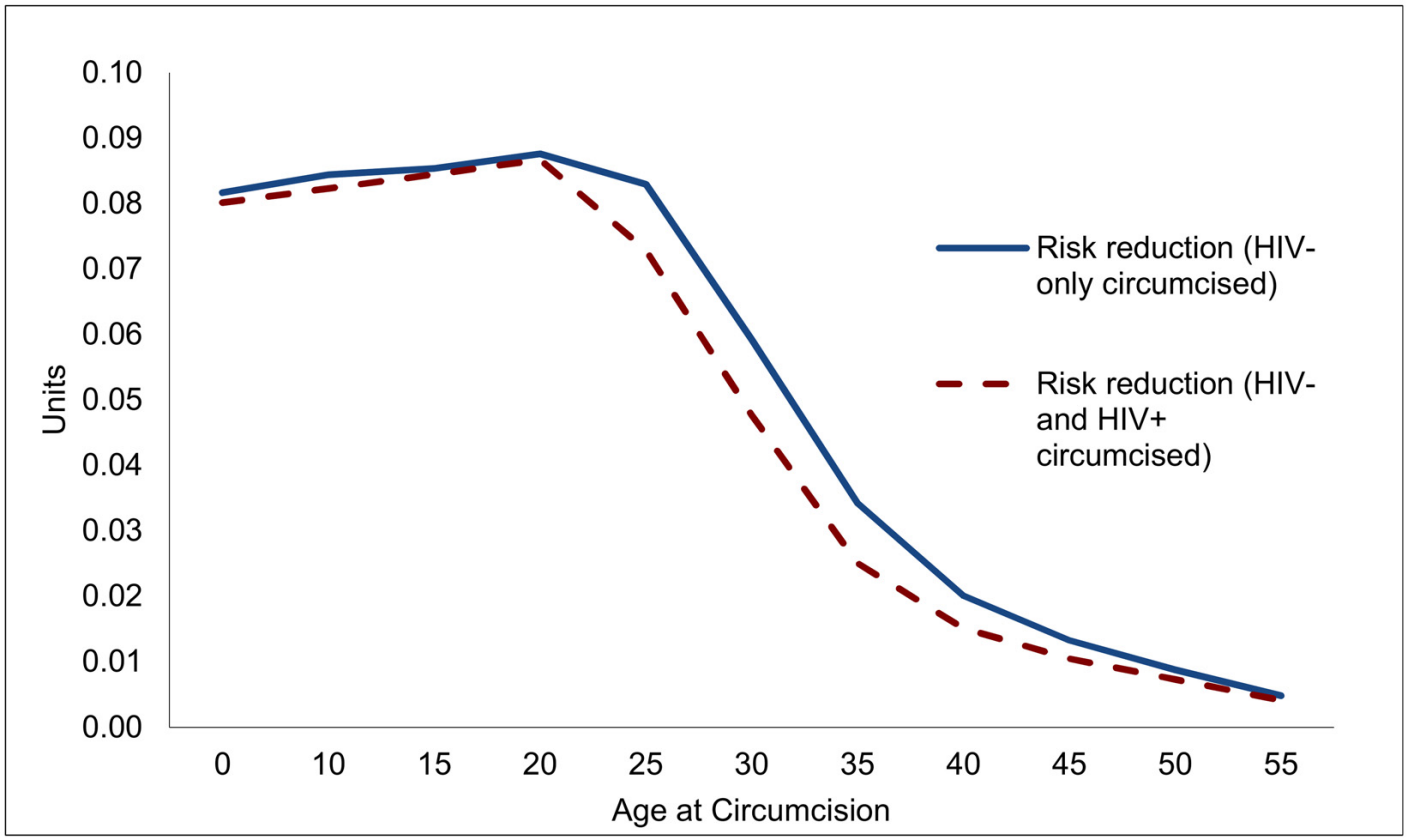
Reduction in lifetime risk of contracting HIV for individual circumcised, by age at circumcision (units).

Overall, each male circumcision averts about 0.09 HIV infections over the course of a circumcised individual’s life for circumcisions at ages 15 or 20 (Fig 2), but the effect is diminished to 0.06 for individuals at age 30, and only 0.02 for individuals circumcised at age 40. While the principal determinant of the effect of a male circumcision is the life cycle of the individual risk of contracting HIV, the evolving state of the epidemic also plays a role. For this reason, the lifetime risk of contracting HIV and the effect of VMMC are somewhat diminished for circumcisions occurring at ages 15, 10, or 0.

To determine the effectiveness of VMMC in reducing new infections, and to guide the design of VMMC programs, it is important to know how many men who might undergo VMMC are HIV-positive already, and how this might compromise the effectiveness of VMMC. If HIV prevalence among males undergoing VMMC is the same as for their cohort overall, the effects of VMMC are not much diminished at young ages (up to age 20), but decline by 10% by age 25 and by over a quarter for circumcisions at ages 35 or 40.

The HIV incidence-reduction effects of VMMC, though, are larger than the direct effects (on individuals circumcised), because individuals not infected also do not transmit HIV. The indirect effects could potentially be large because of relatively high rates of male-to-female transmission of HIV (in 2014, 55% of estimated adult HIV infections in South Africa occurred among women [31]), and female sexual partners not infected as an indirect result of VMMC also do not transmit HIV to infants or their other current or subsequent sexual partners.

**Fig 3:**
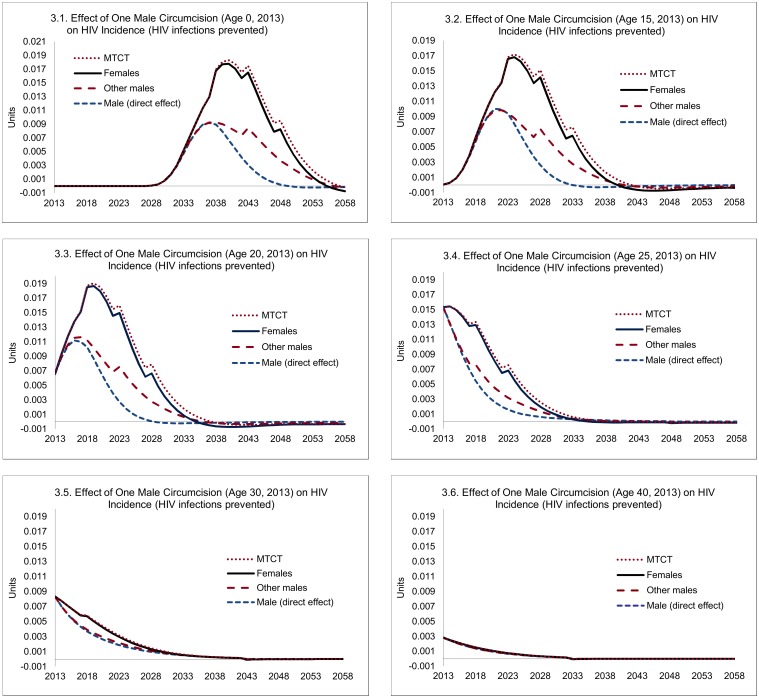
Direct and indirect effects of one male circumcision on HIV incidence. Note: "Male (direct effect)" shows the expected effect on the individual circumcised, "Females" shows the population-level effects among female partners, "Other males" represent population-level effects among males other than the individual circumcised, and "MTCT" stands for HIV infections averted through mother-to-child transmission.

The magnitude of such indirect effects is illustrated in Fig 3. Three lessons can be drawn. First, the magnitude of the indirect effects is substantial, especially if circumcisions occur at young ages, where they exceed the direct effects. Second, as can be anticipated, the indirect effects occur later—HIV infections averted among women occur only after the direct effects have started to accumulate, and HIV infections averted among other men or infants depend on HIV infections averted among women. For example, for individuals circumcised at age 20, the median time to HIV infections averted is 5 y, but for the indirect effects it is 10 y. Third, the indirect effects of circumcisions decline steeply with age—a consequence of declining sexual activity.

**Fig 4:**
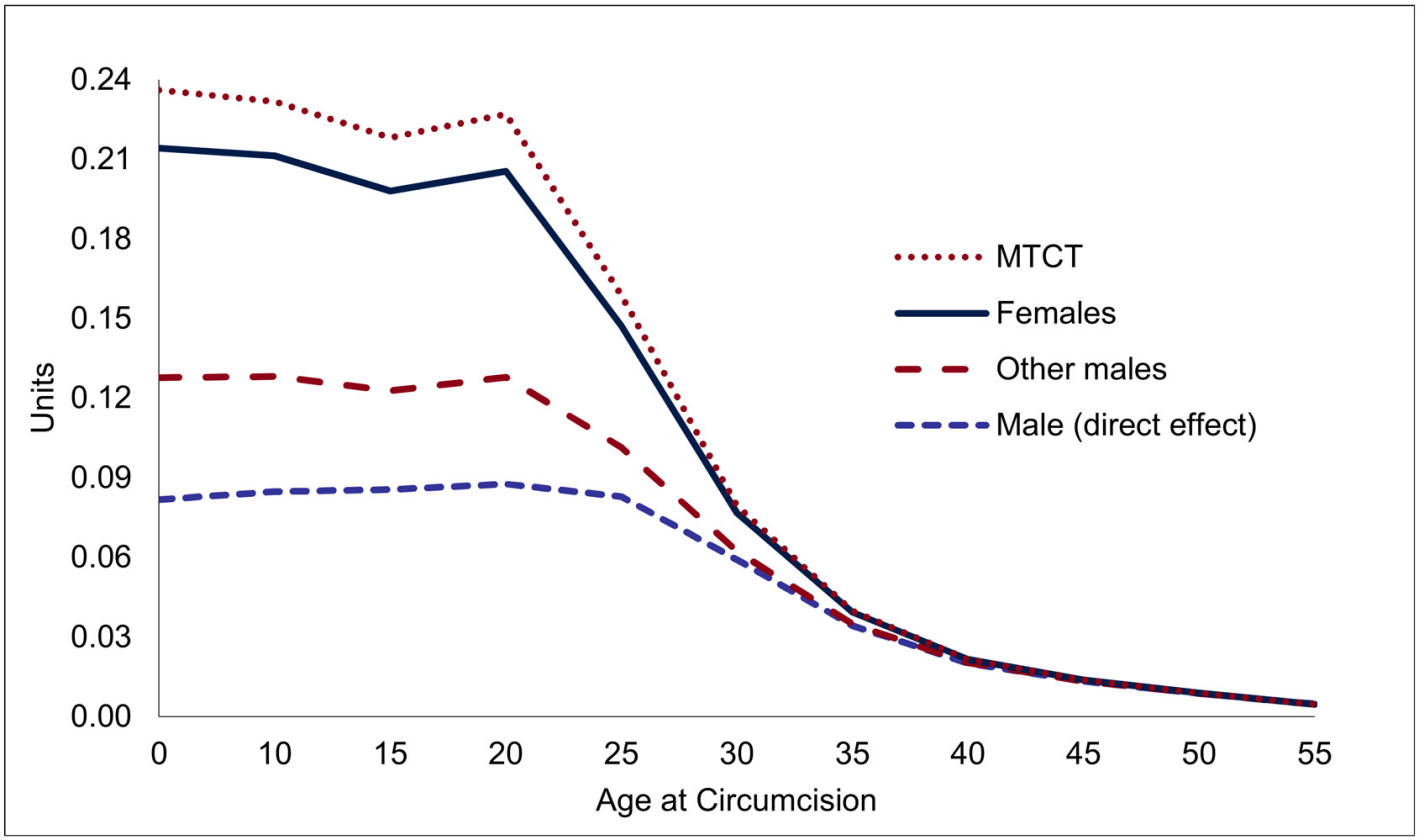
Accumulated effect of one male circumcision on HIV incidence, by age of individual circumcised (HIV infections prevented, in units). Note: Lines are stacked, so that the distance between the lines gives the effect for each group, and the highest line shows the total effect.

Overall, each male circumcision for young adults (age 20) averts 0.23 HIV infections, of which 0.09 can be attributed to the direct effects and 0.14 HIV infections to the indirect effects (Fig 4). The total effects decline after age 20—by about one-third by age 25, and two-thirds by age 30, primarily because of the steep decline in the indirect effects (from 0.14 at age 20 to 0.08 by age 25, and to 0.02 by age 30).

### Financial Analysis

**Fig 5:**
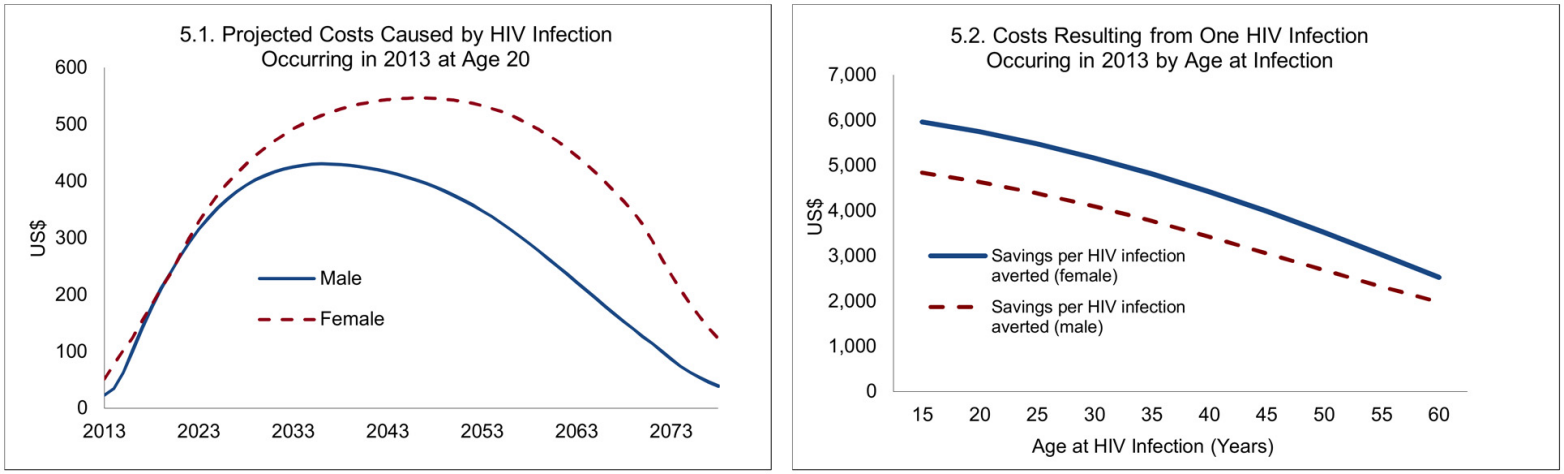
Projected costs caused by one new HIV infection, 2013.

The costs caused by one new HIV infection are illustrated in Fig 5, with detailed projections for a 20-y-old who contracted HIV in 2013, and summary estimates across ages (applying a discount rate of 5%). For a 20-y-old who becomes infected in 2013, the expected costs caused by a new HIV infection are spread over about 60 y and peak at an annual cost of about US$550 for women and US$430 for men. The differences between men and women largely arise because of longer survival for women—projected at 37 y for women and 28 y for men, reflecting both longer underlying (non-AIDS) survival of women and somewhat lower HIV-related mortality rates for women. Overall, the projected costs reflect the prospect of moving on to treatment, survival with and without treatment, transition into second-line treatment, and an increase in unit costs (other than drugs) in line with GDP per capita. Because of the role of survival, the costs caused by HIV infections differ by age at infection. The remaining life span of an individual who becomes infected at age 40 or 60 is obviously much lower than for an individual infected at age 20, and the costs caused by new HIV infections therefore decline with age at infection. This point is relevant for the savings resulting from male circumcision, because VMMC later in life averts not only fewer HIV infections but the financial savings from each HIV infection averted are lower.

Results on the cost-effectiveness of male circumcision follow from the estimates on the effectiveness and the unit costs of VMMC. If one adult VMMC costs US$104, then circumcisions between ages 10 and 20 avert HIV infections at a cost of US$450 to US$478 per infection averted (Table 1). With declining effectiveness of VMMC at higher ages, the cost-effectiveness also deteriorates steeply, resulting in a cost per HIV infection averted of US$1,300, US$4,800, and US$11,800 for individuals circumcised at ages 30, 40, and 50, respectively. Infant circumcision, at a unit cost of US$52 and similar effectiveness, is the most cost-effective in terms of the reduction in the lifetime risk of contracting HIV, at US$221 per HIV infection averted, although these results arrive with a long lag. If HIV infections averted are discounted at a rate of 5%, early infant male circumcision (EIMC) is therefore about as cost-effective as circumcisions at age 15 (Table 1).

**Table 1:** Summary of results (based on one male circumcision performed in South Africa in 2013).

Age at male circumcision	Impact on HIV Incidence			Male circumcisions per HIV infection averted	Cost per HIV infection averted (US$)		Net savings from one male circumcision (US$, at 5% disc.)	Amortization period (y, at 5% disc.)	Financial rate of return (%)
	Total	Direct	Indirect		(at 0% disc.)	(at 5% disc.)			
0	0.236	0.082	0.154	4.2	221	859	301	30.6	7.8
10	0.232	0.084	0.147	4.3	450	1,157	401	22.8	9.9
15	0.218	0.085	0.133	4.6	478	867	475	17.4	11.5
20	0.227	0.088	0.139	4.4	460	659	617	12.2	14.5
25	0.159	0.083	0.076	6.3	657	854	412	11.6	13.6
30	0.079	0.059	0.020	12.6	1,316	1,749	123	16.6	8.8
35	0.040	0.034	0.006	25.1	2,623	3,455	−2	n.a.	4.9
40	0.022	0.020	0.002	46.2	4,826	6,135	−52	n.a.	1.8
45	0.014	0.013	0.000	72.7	7,592	9,268	−74	n.a.	−0.9
50	0.009	0.009	0.000	113.3	11,819	13,709	−86	n.a.	−3.6
55	0.005	0.005	0.000	214.2	22,350	24,157	−95	n.a.	−6.8

These estimates point to the potential of male circumcision to contain the costs of the HIV/AIDS response and contribute to financial sustainability of the HIV response: the one-off costs per HIV infection averted through VMMC at younger ages are of a similar magnitude as the annual cost of treatment (between US$267 and US$531 as of 2014). Considering that people living with HIV in South Africa (provided they initiate treatment early) can have a near-normal life expectancy [32], including several decades of receiving treatment, the cost savings from VMMC thus can prove to be many times higher than the costs of the procedure.

**Fig 6:**
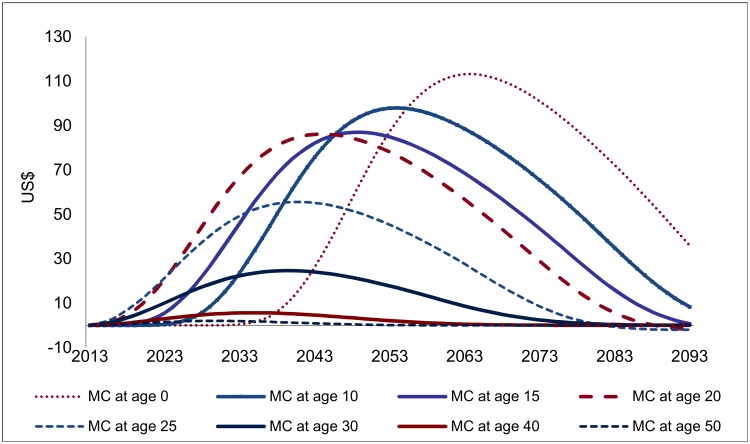
Projected financial savings following one male circumcision (USD, adjusted for inflation).

However, because the impact of VMMC on HIV incidence is distributed over a long period (about 20 y for young adults, but somewhat less at older ages; see Fig 3), and the expected costs caused by new HIV infections also extend over a long period (e.g., about 60 y for an individual infected at age 20; Fig 5.1), the financial savings as a consequence of one circumcision at different ages are spread over extremely long periods for the purpose of policy analysis, as shown in Fig 6, which extends over 80 y.

For male circumcisions occurring above age 20, the relative magnitude of the projected savings from one VMMC (Fig 6) largely mirrors the findings on the effectiveness of VMMC. The consequences of lower remaining life expectancy at higher ages are visible; for circumcisions occurring at higher ages, the financial savings are distributed over a shorter period and decline more than proportionally compared to the effectiveness of VMMC. For circumcisions below age 20, any effects occur later, especially for infant circumcision, for which the cost savings peak only 50 y after the circumcision is performed. The cost savings also come out higher for circumcisions occurring at young ages. This is a consequence of accounting for increasing costs as gross domestic product (GDP) per capita rises, which—over several decades—makes a large difference.

However, for any discount rates commonly applied in policy analysis, the longer lags mean that the financial savings for circumcisions at young ages carry a lower weight. If a discount rate of 5% is applied, the financial savings from infant circumcision are about one-half lower than for circumcisions at age 20, even though the undiscounted cost savings are 20% higher.

**Fig 7:**
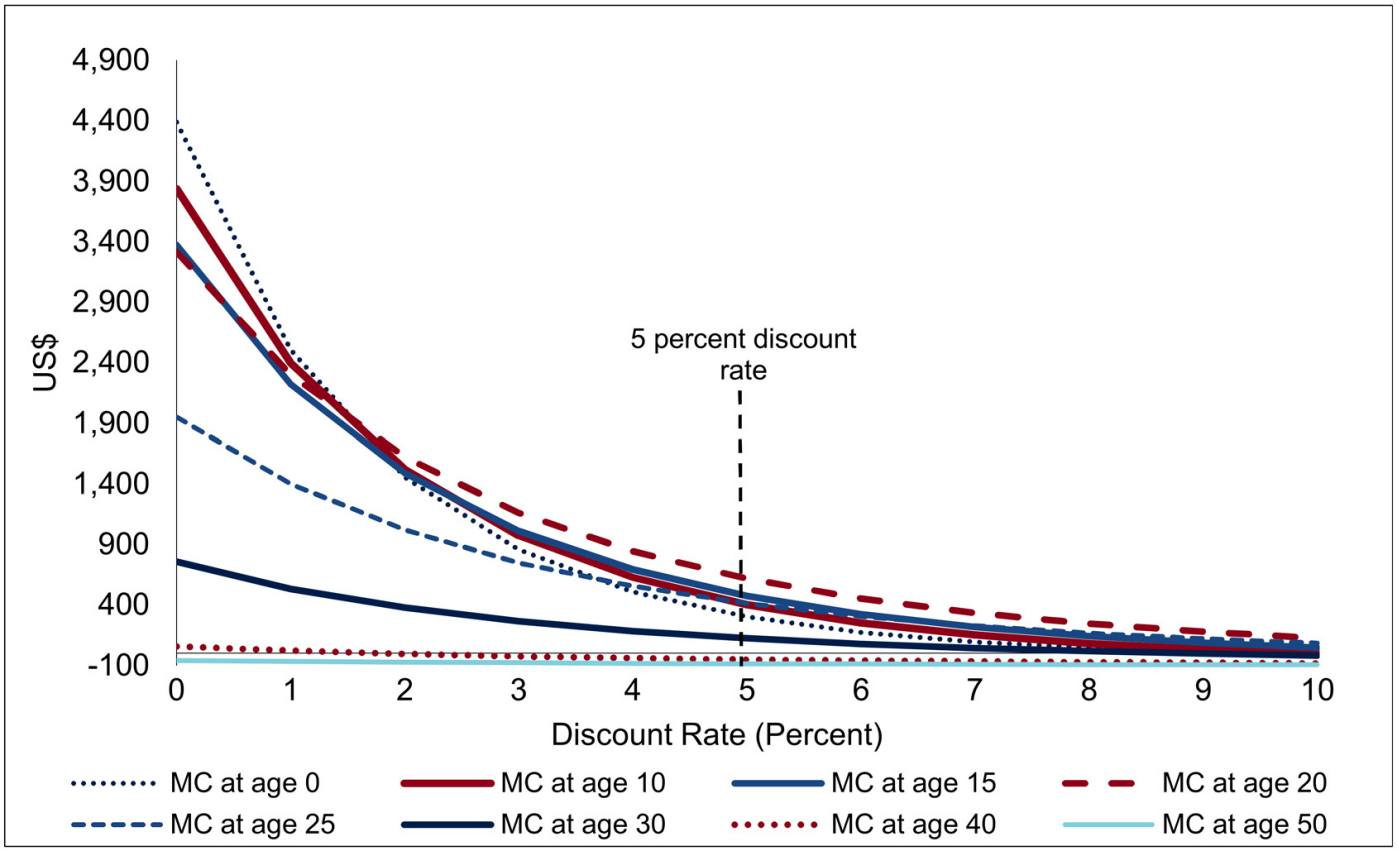
Net savings from one male circumcision, by discount rate (USD, adjusted for inflation).

Indeed, owing to the extremely long period over which the effects are spread, the discount rate applied becomes one of the most important factors in interpreting the financial savings (Fig 7). For a discount rate of 5%, the net savings from one VMMC (the present discounted value of the savings shown in Fig 6, minus the costs of one VMMC) are largest for VMMCs performed at age 20 (at US$617), followed by VMMC performed at age 15 (US$475). At age 30, the net financial savings are already much diminished (US$123), and they drop to zero from about age 35. If the discount rate is at 3% rather than 5%, the net savings from one VMMC at age 20 come out at US$1,010 instead of US$475, and at a discount rate of 7%, they shrink to US$218. Owing to the long time lags involved, results on EIMC and circumcision of adolescents are the most sensitive to the discount rate applied—while EIMC is the most or among the most cost-effective type of male circumcision for discount rates below 2%, it is less effective (in terms of net savings from one male circumcision) than VMMC at ages 10 to 25 for a discount rate of 4% and higher.

This sensitivity analysis with respect to the discount rate also offers insights on the robustness of findings with respect to treatment costs (the dominant factor in financial savings). If unit costs of treatment gradually decline by one-half by 2050, i.e., by about 2% annually, the implications are very similar to an increase in the discount rate of two percentage points—net savings are much diminished, but results on the cost-effectiveness of VMMC overall and the role of age at circumcision come out similarly.

Nevertheless, the thrust of the findings—that VMMC generates very substantial financial savings which more than offset the costs of VMMC—is robust to changes in the discount rate. For VMMC at ages 20 or 25, the costs of VMMC are amortized (i.e., fully refinanced by the resulting savings) after only 12 y; over this period, the discount rate does not play a dominant role (Table 1). An alternative way of addressing the sensitivity of the results with respect to the interest rate is calculating the financial rate of return: at which interest rate are the net savings from one VMMC, discounted at that interest rate, equal to zero, or up to which interest rate are investments in VMMC good financial investments, yielding a financial return in excess of that interest rate? Indeed, investments in VMMC emerge as good financial investments (even before evaluating the health outcomes): VMMCs performed between ages 10 and 25 yield a financial rate of return between 9.9% and 14.5%. Even infant circumcisions—in spite of the long lags—carry a respectable financial rate of return of 7.8%.

### Summary of Results

VMMC in South Africa is an intervention that is highly effective in preventing HIV infections, and also a very good financial investment, with savings far outweighing the costs for circumcisions at most ages. Each male circumcision averts up to 0.23 HIV infections for young adults (up to age 20), but the effect declines for VMMCs performed at higher ages, as the reduction in the lifetime risk of contracting HIV for the individual circumcised diminishes, and even more so because the indirect effects decline steeply with increasing age at circumcision. For circumcisions at younger ages, the effect is similar to circumcisions at age 20, but the effects occur with a long delay, especially for EIMC.

In terms of financial returns, circumcisions at age 20 are most effective, with a financial rate of return of 14.5% (Table 1) or resulting in net savings of US$617 when a discount rate of 5% is applied. While the financial savings are distributed over extremely long periods (Fig 6), the large magnitude of the financial savings means that the initial costs are amortized in about 12 y for VMMCs in young adults. Infant circumcision—about as effective as VMMC for young adults, but less expensive—is more cost-effective than circumcision for young adults. However, as the effects occur with a long delay, the net savings and financial returns are much lower than for young adults.

## Discussion

In contrast to other studies, which analyze and compare various population-level scenarios, we conducted an incremental analysis to determine the expected effects of circumcising one male individual at a specific age. Our analysis thus did not provide estimates on how a VMMC policy contributes to attaining national goals on reducing HIV incidence on the population-level over some policy period. Instead, our approach yields more precise estimates of the effects and cost-effectiveness of VMMC and provides direct estimates of the consequences of current policies under consideration.

The improved precision is related to the long time frames (VMMC to HIV infections averted, and costs caused by new HIV infections which also extend over decades). National policy scenarios address this problem by adopting long time horizons (now frequently up to 2050), but produce imprecise estimates of cost-effectiveness as inputs and outcomes are aggregated over long periods and are subject to bias (especially for circumcisions occurring late in the policy period, the consequences are barely captured). The incremental analysis offered here works around these problems by considering the effects of a policy at a specific point of time only, which then allows us to track the consequences of this policy as long as necessary to capture the full effects (Fig 6). Because the long lags regard especially the financial savings resulting from VMMC, our approach is particularly suitable to capture the financial returns to investments in VMMC.

In terms of the quantitative results, the findings from our analysis on the cost-effectiveness of VMMC are broadly consistent with those of other studies on VMMC in general or across various age groups: VMMC is a highly effective HIV prevention intervention in a country with a generalized HIV epidemic and under many circumstances is cost-saving as the financial savings owing to reduced HIV incidence outweigh the costs of providing VMMC. VMMC is most effective at about age 20, declines at higher ages because of the diminished direct and indirect effects on HIV incidence. For VMMC at lower ages, the lifetime impact on HIV infections averted is about the same as for young adults. However, because of the delayed impact (especially for EIMC), the effects of circumcising infants or young adolescents are diminished relative to young adults when some discounting is applied to the outcomes.

With regard to informing policies, the relevance of our approach derives from the ability to make precise statements of the effects of policies considered under the government’s current budget process, i.e., typically over a one-year period. In this regard, it does not only offer estimates of the health returns to investments in VMMC but also of the implications of current policies in terms of the fiscal space absorbed by the HIV/AIDS response—what are the net costs or net savings over the coming years associated with current spending on VMMC, and—if there are net savings—how quickly are the current outlays refinanced by anticipated savings?

More fundamentally, the approach also offers lessons relevant for the design of the HIV/AIDS program. What is the contribution of current or additional investments in VMMC to the long-term costs and the financial sustainability of the HIV/AIDS response? Can outcomes or long-term costs be improved by reallocating current funds between interventions [33]? How much should be invested in creating additional demand for VMMC to maximize its potential in terms of controlling the epidemic and reduce long-term costs?

To some extent, the analysis and the magnitude of the estimated effects of VMMC is specific to South Africa. The magnitude of the financial savings, however, suggests that VMMC would save costs at a much lower level of HIV prevalence than observed in South Africa, at least for some age groups. Our analysis identified some aspects of the cost-effectiveness of VMMC, which are plausibly relevant more generally, notably the decline in the effectiveness of VMMC by age, reflecting three factors: (1) at higher ages, controlling for behavior, the remaining lifetime risk of contracting HIV is lower; (2) a sorting effect: individuals adopting high-risk behavior are more likely to become infected early, whereas individuals not infected at higher ages are statistically more likely to have adopted and to adopt low-risk sexual behavior; and (3) the role of declining sexual activity and time lags between successive rounds of downstream infections, which explain much of the decline in the indirect effects of VMMC.

The principal limitations of the analysis arise from the long time (decades) over which the effects of VMMC unfold. This is evident from the sensitivity analysis with respect to the discount rate (Fig 7), but the uncertainties regard more fundamentally the future course of the epidemic and of the HIV/AIDS response. With regard to HIV incidence, the uncertainties become more pronounced for circumcisions conducted at young ages—for VMMC occurring at age 25, the impacts on HIV incidence are spread over 15 y; for EIMC, the impact sets in only after about 15 y and is then spread over another 25 y (Fig 7). For the financial returns to investment in VMMC, the uncertainties are compounded by the fact that projected treatment needs to extend over several decades, on top of the lags between VMMC and the impact on HIV incidence.

In terms of the modelling assumptions, perhaps the most significant factor introducing uncertainty to the projections is the assumed zero impact of male circumcision on male-to-female transmission of HIV. If an effect of VMMC on male-to-female transmission of HIV is present, the indirect impact of male circumcision on female sexual partners would come out larger (and could even double by some account [34]). In this case, the effects of VMMC would come out larger than described in this paper, and the decline in the effectiveness at older ages (driven largely by the indirect effects) would be steeper.

## Supporting Information

S1 Analysis Plan Analysis plan. (DOCX)

S1 Checklist CEA checklist. (PDF)

S1 Statement STROBE statement. (PDF)

S1 Text Adapting the ASSA2008 model. (DOCX)

## Abbreviations

EIMC: early infant male circumcision
GDP: gross domestic product
PEPFAR: President’s Emergency Plan for AIDS Relief
UNAIDS: Joint United Nations Programme on HIV/AIDS
USAID: US Agency for International Development
VMMC: voluntary medical male circumcision
