# Supplementary material for: Effectiveness of and Financial Returns to Voluntary Medical Male Circumcision for HIV Prevention in South Africa: An Incremental Cost-Effectiveness Analysis
Source: PLoS Med. 2016 May 3;13(5):e1002012. doi: 10.1371/journal.pmed.1002012 (PMC4854479; doi:10.1371/journal.pmed.1002012)
Supplement: S1 Analysis Plan — (DOCX) [file pmed.1002012.s001.docx]

# Effectiveness of Financial Returns to Voluntary Medical Male Circumcision: Analysis Plan

The analysis started out (1) from a perception of shortcomings in the literature on the cost-effectiveness of HIV prevention interventions (and, among these, especially of VMMC) associated with the long time frames typical for HIV transmission and disease progression, and (2) from a recognition that an incremental analysis could address these shortcomings. The ASSA2008 model was selected for the analysis primarily because alternative models were proprietary or closed-source and could not be adapted for the purposes of the analysis. Given the objectives of the analysis, the principal challenge was to adapt the ASSA2008 model for the specific needs of the analysis, while the experiments performed on the model (raising the number of male circumcisions at specific ages by one in 2013, and tracking the impacts) were straightforward and implemented as planned. Reflecting the long times frames required by the analysis, the set of results was expanded beyond the original plans by indicators like the amortization period, a summary indicator of the immediacy of the financial returns to investments in VMMC.

## Objectives of Analysis

HIV/AIDS is characterized by very long time frames of disease progression and – relatedly – disease transmission. Individuals contracting HIV on average survive for about 10 years even without treatment, and for several decades with a high chance of access to treatment that has become the norm in countries facing a serious HIV epidemic. An individual contracting HIV may therefore transmit HIV for decades, and the consequences in terms of the demand for medical services (treatment etc.) are spread out even further (a long period on treatment stacked on top of the lags between primary infection and downstream infections). For VMMC, the challenges arising from these lags are even more pervasive, because of the additional lag between the intervention and the effects in terms of HIV infections averted.

Model-based evaluations of HIV prevention policies therefore tend to adopt a fairly long time horizon of 10 to 20 years, and recent work on the effectiveness of VMMC has frequently applied a time horizon of 35 years (through 2050). With regard to assessing the cost-effectiveness of an HIV prevention intervention, there are two shortcomings of these policy studies:

- Estimating the cost-effectiveness of an HIV prevention intervention based on the ratio of the effects of the intervention and its costs ov*er a long period* gives a very imprecise measure of the cost-effectiveness of *current* HIV prevention interventions (or of interventions at any point in time within the policy period).
- If the time horizon on the HIV prevention interventions considered and the effects is the same, the analysis does not capture the full effects of HIV prevention interventions occurring late within the period considered.

Policy studies in which the policy period coincides with the period over which the effects are evaluated cannot address both of these challenges, as the first calls for a short time horizon, and the latter calls for long time horizons.

**The primary objective of the analysis is to offer precise estimates of the cost-effectiveness of VMMC** which address these two challenges. This is achieved by (1) estimating the cost-effectiveness of an intervention which occurs in a specific year; and (2) use the model to project the effects of the interventions until they dissipate, i.e., not imposing a cut-off date ex ante. This means that this “incremental” analysis is not a fundamental alternative to policy studies over some longer period – projecting the consequences of one VMMC requires a fully specified epidemiological model and implies assumptions on policies over the evaluation period. Instead, it provides a framework for utilizing an epidemiological and policy model specified *over a long period* for the analysis of *current* HIV prevention interventions. **A secondary objective of the analysis is to estimate the effects of VMMC by age,** in sync with more recent approached to assessing the effectiveness of VMMC policies.

## Choice and Adaptation of Model

The ASSA2008 model, the latest version of the ASSA model released in 2011, was chosen for the analysis for several reasons.

- The model is programmed in MS Excel so the relevant code is open-source and can be adapted for the purposes of the present analysis.
- The age structure of the population (modelled in one-year cohorts) is granulated sufficiently to capture the dynamics of VMMC and allow an analysis of the effectiveness of VMMC by age. In some alternative models, age cohorts extend over 5 or more years, which introduces some churning with regard to the effects of VMMC over time or by age at circumcision.
- The model was developed and has had a high acceptance in South Africa, one of the most important countries from a VMMC policy perspective.

To allow the intended analysis, though, the model needed to be adapted in three directions:

- The ASSA 2008 model, released (in 2011) at about the beginning of the drive to expand VMMC across Southern Africa, does not explicitly account for the effects of VMMC. The model therefore needed to be adapted by differentiating between uncircumcised and circumcised men. Using the existing structure of the model for males for uncircumcised men, relevant arrays then had to be duplicated to also account for circumcised men, and some assumptions needed to be added on the historical uptake of VMMC to approximate the population-level prevalence of male circumcision. Further, the parameters describing female-to-male transmission of HIV needed to be adapted, and the risk of contracting HIV had to be calculated separately for circumcised and uncircumcised males.
- Some structure needed to be added to carve out the effects of one *additional* male circumcision. This required adding some structure to increase the number of males getting circumcised at a specific age in a specific year (2013 for the purpose of this analysis) by one. Because the analysis is geared at tracking the effect of one *additional* male circumcision over time, it is also necessary to neutralize population-level effects – e.g., adding some structure to ensure that the individual additionally circumcised at some age would not otherwise become circumcised at a later age.
- It is a well-known shortcoming of the ASSA2008 model that it overestimates mortality among people living with HIV and receiving treatment. The costs caused by an HIV infection were therefore estimated and projected using a module (also programed in MS Excel) with disease progression derived from “Spectrum” output, building on (and updating) work projecting the costs of the South African National Strategic Plan on HIV, STIs and TB 2012-2016 and related cost projections of the costs caused by new HIV infections.

## Changes to Analysis in the Course of the Work

The analysis was implemented as intended at the outset, and the analysis plan has not been changed in response to initial results. However, the presentation of the results has been adapted to take account of the exorbitantly long periods over which the cost savings resulting from one VMMC are spread (the relevant figure cover a time horizon of 80 years). In addition to the discussion of the magnitude of the financial savings (characterized by a high degree of uncertainty in light of the long time period over which they are spread, and highly sensitive to the discount rate), the analysis now also presents estimates of how fast the costs of VMMC are refinanced by subsequent savings (amortization period).
