# Supplementary material for: Effectiveness of and Financial Returns to Voluntary Medical Male Circumcision for HIV Prevention in South Africa: An Incremental Cost-Effectiveness Analysis
Source: PLoS Med. 2016 May 3;13(5):e1002012. doi: 10.1371/journal.pmed.1002012 (PMC4854479; doi:10.1371/journal.pmed.1002012)
Supplement: S1 Checklist — (PDF) [file pmed.1002012.s002.pdf]

Item	Reference	Comment/Summary
**Framework**		
Background of the problem	Introduction, paragraphs 1-5	"Empirical studies and population-level policy simulations show the importance of voluntary medical male circumcision (VMMC) in generalized [HIV] epidemics"; discussion of randomized control trials and other academic literature; discussion of trends in uptake and experiences from scale-up; discussion of previous efforts in improving the efficiency of VMMC services; challenges for modeling and policy analysis arising from long time frames of HIV transmission and disease progression.
General framing and design of the analysis	Introduction, paragraphs 6-9	*Incremental* approach: "Rather than assessing the overall costs and impacts of a VMMC policy scenario (specifying the number of circumcisions at different ages performed over some policy period), it estimates the impacts, over time and across the population, of circumcising one male individual, at a specific age, in a specific year."
Target population for intervention	Methods, paragraph 7	Male population in South Africa, at age 0 and in 5-year increments from age 10 to age 55
Other program descriptors (eg, care setting, model of delivery, timing of intervention)	Methods, paragraph 7	One additional VMMC in 2013 at a specific age
Description of comparator programs	Methods, paragraph 7	Baseline without the additional VMMC
Boundaries of the analysis		Analysis of effects of VMMC on HIV incidence (infections averted, direct and indirect (by population group)) and cost-savings owing to the reduced incidence
Time horizon		2013-2093 to capture the full financial effects (lifetime costs of treatment), although the expected effect of VMMC on HIV incidence materialize over a shorter perid (about 10-40 years, depending on the age at circumcision).
Statement of the perspective of the analysis		Sectoral (health system, more specifically HIV/AIDS response)
**Data and Methods**		
Description of event pathway		See "Description of model used" (I and II)
Identification of outcomes of interest in analysis		Impact of one VMMC on HIV incidence over time and across the population; financial savings owing to reduced HIV incidence
Description of model used (I: demographic and epidemiological)	Methods, paragraphs 2-3	ASSA2008 national ("lite") model, adapted to capture the effects of male circumcision
Description of model used (II: costing)	Methods, paragraphs 4-5	Disease progression model in line with Spectrum estimates for South Africa and treatment coverage targets from the "National Strategic Plan on HIV, STIs and TB 2012-2016"
Modeling assumptions (I: demographic and epidemiological)	Methods, paragraph 3	Female-to-male HIV transmission rate for circumcised males 60 percent lower than for uncircumcised males (in accordance with WHO/UNAIDS data)
Modeling assumptions (II: costing)	Methods, paragraph 5	Costs of treatment assumed in line with recent South African Investment Case; other cost items from earlier estimates, adjusted for inflation; all costs (except drugs) assumed to grow at a rate of one percent annually (approximately the rate of growth of GDP per capita); mark-up of 5.5 percent for overhead expenses. Costs of VMMC in line with most recent estimates (2015) used in South African "HIV investment case."
Diagram of event pathway (model)	not applicable	not applicable; assumptions of software used for epidemiological projections is documented in the relevant publications
Software used		ASSA2008 model, Spectrum, Excel
Complete description of estimates of effectiveness, resource use, unit costs, health states, and quality-of-life weights and their sources	Methods, paragraph 5	See "Modeling assumptions (II: costing)"
Methods for obtaining estimates of effectiveness, costs, and preferences	Methods, paragraph 5	See "Modeling assumptions (II: costing)"
Critique of data quality		As the analysis builds on a model estimating and projecting the state of HIV/AIDS in South Africa, a principal aspect of data quality is how well the model matches available data. In a recent evaluation of various models describing and projecting the state of HIV/AIDS in South Africa (Eaton and others, *The Lancet Global Health* , October 2025), the ASSA2008 model used in the analysis performs relatively well compared to some alternative models.
Statement of year of costs	Methods, paragraph 5	Cost estimates of treatment costs current as of March 2015, other cost items from earlier estimates (2010, 2012), adjusted for inflation
Statement of method used to adjust costs for inflation		Where current prices were unavailable, earlier estimates were adjusted for inflation to obtain estimates at 2013 prices. For the projections, most costs are assumed to grow at about the same rate as real GDP per capita (i.e., without inflation) to account for increasing *real* wages.
Statement of type of currency		US$ for all results; cost estimates orignially in ZAR were transformed into US$ applying a year-average exchange rate.
Source and methods for obtaining expert judgment	not applicable	not applicable
Statement of discount rates		The main results are presented for a discount rate of 5 percent; sensitivity analysis with regard to the interest rate
**Results**		
Results of model validation		As the analysis draws on a model that is readily available in the public domain, a model validation was not conducted. However, the model appears in a recent "validation analysis of ten mathematical models of HIV epidemiology in the antiretroviral therapy era" (Eaton and others, 2015)*, in which the model tended to perform better than some alternative models in projecting national HIV prevalence trends.<br>* Eaton, Jeffrey W., and others, 2015, "Assessment of epidemic projections using recent HIV survey data in South Africa: a validation analysis of ten mathematical models of HIV epidemiology in the antiretroviral therapy era," *The Lancet Global Health* , Volume 3, No. 10 (October 2015), e598–e608.
Reference case results (discounted at 3% and undiscounted): total costs and effectiveness, incremental costs and effectiveness, and incremental cost-effectiveness ratios	Table 1	Table 1 (discounted at 5 percent)
Results of sensitivity analyses	Figure 7	Figure 7 (sensitivy analysis with regard to the discount rate applied)
Other estimates of uncertainty, if available	not applicable	not applicable
Graphical representation of cost-effectiveness results	Table 1	Table 1 (discounted at 5 percent)
Aggregate cost and effectiveness information	Table 1	Table 1 (discounted at 5 percent)

Item	Reference	Comment/Summary
Disaggregated results, as relevant	Figures 5-6	Figure 5 (projected costs of one new HIV infection in 2013 over time), Figure 6 (projected financial savings from one VMMC by age at circumcision) and accompanying text
Secondary analyses using 5% discount rate	not applicable	not applicable; main analysis applies a discount rate of 5 percent
Other secondary analyses, as relevant	Figure 1-4	Figures 1-4 (demographic and epidemiological effects of one VMMC with accompanying text)
**Discussion**		
Summary of reference case results	Results, paragraphs 18-19	VMMC in South Africa is highly effective in preventing HIV infections, and also a very good financial investment, with savings far outweighing the costs for circumcisions at most ages. Each male circumcision averts up to 0.23 HIV infections for young adults, but the effect declines for VMMCs performed at higher ages. For circumcisions at younger ages, the effect is similar to circumcisons at age 20, but the effects occur with a long delay. In terms of financial returns, circumcisions at age 20 are most effective, with a financial rate of return of 14.5 percent. While the financial savings are distributed over extremely long periods, the large magnitude of the financial savings means that the initial costs are amortized in about 12 years for VMMCs in young adults. Infant circumcision–about as effective as VMMC for young adults, but less expensive–is more cost-effective than circumcision for young adults. However, as the effects occur with a long delay, the net savings and financial returns are much lower than for young adults.
Summary of sensitivity of results to assumptions and uncertainties in the analysis	Discussion, paragraphs 7-8	Assumed zero impact of male circumcision on male-to-female transmission of HIV constitutes most significant factor introducing uncertainty to the projections.
Discussion of analysis assumptions having important ethical implications		Not applicable.
Limitations of the study	Discussion, paragraphs 7-8	Principal limitations arise from the long time over which effects of VMMC unfold: see sensitivity analysis of the discount rate applied; general uncertainty about the future course of the HIV epidemic and the HIV/AIDS response.
Relevance of study results for specific policy questions or decisions	Discussion, paragraphs 1-5	With regard to informing policies, the relevance of our approach derives from the ability to make precise statements of the effects of policies considered under the government's current budget process, i.e., typically over a one-year period. In contrast, most alternative studies only offer average effects of a policy pursued over several decades. Substantially, the analysis does not only offer estimates of the health returns to investments in VMMC, but also of the implications of current policies in terms of the fiscal space absorbed by the HIV/AIDS response.
Results of related cost-effectiveness analyses	Discussion, paragraph 3	Findings are broadly consistent with related CEA.
Distributive implications of an intervention	Not applicable	Not applicable
