# Supplementary material for: Effectiveness of and Financial Returns to Voluntary Medical Male Circumcision for HIV Prevention in South Africa: An Incremental Cost-Effectiveness Analysis
Source: PLoS Med. 2016 May 3;13(5):e1002012. doi: 10.1371/journal.pmed.1002012 (PMC4854479; doi:10.1371/journal.pmed.1002012)
Supplement: S1 Statement — (PDF) [file pmed.1002012.s003.pdf]

Item	Item No.	Reference	Comments
**Title and abstract**	1a		Effectiveness of and Financial Returns to Voluntary Medical Male Circumcision for HIV Prevention in South Africa: An **Incremental Cost-Effectiveness Analysis**
	1b		
**Introduction**			
Background/rationale	2	Introduction, paragraphs 1-5	"Empirical studies and population-level policy simulations show the importance of voluntary medical male circumcision (VMMC) in generalized [HIV] epidemics"; discussion of randomized control trials and other academic literature; discussion of trends in uptake and experiences from scale-up; discussion of previous efforts in improving the efficiency of VMMC services.
Objectives	3	Introduction, paragraphs 6-9	"Rather than assessing the overall costs and impacts of a VMMC policy scenario (specifying the number of circumcisions at different ages performed over some policy period), it estimates the impacts, over time and across the population, of circumcising one male individual, at a specific age, in a specific year."
**Methods**			
Study design	4	Methods, paragraphs 1-8	extensive presentation of methodology, assumptions, and specific procedures
Setting	5		modeling study, South Africa
Participants	6a	not applicable	not applicable, modeling study
	6b	not applicable	not applicable, modeling study
Variables	7		Impact of one VMMC on HIV incidence over time and across the population; financial savings owing to reduced HIV incidence
Data sources/measurement	8	Methods, paragraph 5	sources for assumptions on costs; also see "Study design"
Bias	9		The analysis was designed to address and remove one important source of bias in estimating the effectiveness and cost-effectiveness of VMMC, arising from arbitrary cut-offs in the evaluation of the impacts. We are not aware of other aspects of the analysis which could introduce systematic bias.
Study size	10	not applicable	not applicable, modeling study
Quantitative variables	11	not applicable	not applicable, modeling study
Statistical methods	12a	not applicable	not applicable, modeling study
	12b	not applicable	not applicable, modeling study
	12c	not applicable	not applicable, modeling study
	12d	not applicable	not applicable, modeling study
	12e		The analysis headlines the sensitivity of the effectiveness and cost-effeectiveness of VMMC for HIV prevention by age at circumcision. The sensitivity of findings is discussed with respect to the extent to which HIV-positive men, as well as HIV-negative men, obtain VMMC, and with respect to the discount rate.
**Results**			
Participants	13a	not applicable	not applicable, modeling study
	13b	not applicable	not applicable, modeling study
	13c	not applicable	not applicable, modeling study
Descriptive data	14a	not applicable	not applicable, modeling study
	14b	not applicable	not applicable, modeling study
	14c	not applicable	not applicable, modeling study
Outcome data	15	not applicable	not applicable, modeling study
Main results	16a	Table 1, Figures 1-6	Table 1 (summary of main results), see Figures 1-4 (demographic and epidemiological effects of one VMMC), Figure 5 (projected costs of one new HIV infection in 2013 over time), Figure 6 (projected financial savings from one VMMC by age at circumcision) and accompanying commentary
	16b	not applicable	
	16c	not applicable	
Other analyses	17	Figure 7	Figure 7 (sensitivity analysis with regard to the discount rate applied)
**Discussion**			
Key results	18	Results, paragraphs 18-19 and Table 1	VMMC in South Africa is highly effective in preventing HIV infections, and also a very good financial investment, with savings far outweighing the costs for circumcisions at most ages. Each male circumcision averts up to 0.23 HIV infections for young adults, but the effect declines for VMMCs performed at higher ages. For circumcisions at younger ages, the effect is similar to circumcisons at age 20, but the effects occur with a long delay. In terms of financial returns, circumcisions at age 20 are most effective, with a financial rate of return of 14.5 percent. While the financial savings are distributed over extremely long periods, the large magnitude of the financial savings means that the initial costs are amortized in about 12 years for VMMCs in young adults. Infant circumcision–about as effective as VMMC for young adults, but less expensive–is more cost-effective than circumcision for young adults. However, as the effects occur with a long delay, the net savings and financial returns are much lower than for young adults.
Limitations	19	Discussion, paragraphs 6-7	principal limitations arise from the long time over which effects of VMMC unfold: see sensitivity analysis of the discount rate applied; general uncertainty about the future course of the HIV epidemic and the HIV/AIDS response
Interpretation	20	Discussion, paragraphs 3-5	In contrast to other studies, which analyze and compare various population-level scenarios, we conducted an incremental analysis to determine the expected effects of circumcising one male individual at a specific age, yielding more precise estimates of the effects and cost-effectiveness of VMMC, and providing direct estimates of the consequences of current policies under consideration. In terms of the quantitative results, the findings from our analysis on the cost-effectiveness of VMMC are broadly consistent with those of other studies on VMMC in general or across various age groups. More fundamentally, the approach also offers lessons relevant for the design of the HIV/AIDS program.
Generalisability	21	Discussion, paragraph 6	While the analysis is specific to South Africa (generalized HIV epidemic), the paper identifies aspects of the cost-effectiveness of VMMC which are relevant more generally, noteably the decline in the effectiveness by age at circumcision; the study suggests that VMMC may be cost-saving at much lower level of HIV prevalence, at least for some age groups
**Other information**			
Funding	22	Metadata	The analysis was funded by the World Bank.
