# Supplementary material for: Effectiveness of and Financial Returns to Voluntary Medical Male Circumcision for HIV Prevention in South Africa: An Incremental Cost-Effectiveness Analysis
Source: PLoS Med. 2016 May 3;13(5):e1002012. doi: 10.1371/journal.pmed.1002012 (PMC4854479; doi:10.1371/journal.pmed.1002012)
Supplement: S1 Text — (DOCX) [file pmed.1002012.s004.docx]

# Supporting Information

# (S1 Text) Adapting the ASSA2008 Model

The ASSA2008 model is the latest edition of the demographic and epidemiological model developed and maintained by the Actuarial Society of South Africa (ASSA). The model has two versions: the “full” model, which divides the population by ethnic group (“African,” “Coloured,” “Indian,” and “White”) and by sexual risk behavior, and the “lite” model, which distinguishes by sexual risk behavior only, but not along ethnic lines. The analysis presented in our paper is based on the “lite” model. The model is freely available from the ASSA website (http://www.actuarialsociety.org.za/Societyactivities/CommitteeActivities/DemographyEpidemiologyCommittee/Models.aspx), and is documented in ASSA (2011) [1].

The published version of the ASSA2008 model does not explicitly account for male circumcision and needed to be adapted. This note explains several features of the model relevant for the analysis (e.g., the different risk groups, which are underlying the “sorting” effects referred to in the analysis), and explains how the model was adapted to account for male circumcision.

In the ASSA2008 model, the population is split into six groups for each sex. The young (up to age 13) and the old (from age 60) are not sexually active or at least cannot acquire HIV. The population of ages 15–59 is split into the groups “PRO” (“individuals whose level of sexual activity is such that it is similar to that of sex workers and the level of condom usage and infection with sexually transmitted diseases (STDs) is similar to that of the STD group”), “STD” (“individuals whose level of sexual activity is such that their HIV prevalence is similar to someone regularly infected with STDs”), “RSK” (“individuals with a lower level of sexual activity, but who are still at risk from HIV in that they have, on average, one new partner per annum and sometimes engage in unprotected sex”), and “NOT,” i.e., “individuals who are not at risk of an HIV infection” (either because they are not sexually active or are in a stable monogamous relationship with an HIV-negative partner). The ASSA2008 model captures heterosexual intercourse only, because this is the dominant mode of HIV transmission in South Africa.

For each risk group, individual sexual activity is characterized by assumptions regarding the number of new partners per year, the number of contacts per partner, and the distribution of partners by risk group (e.g., 60 percent of partners of “RSK” individuals are pulled from the same group, the others from the “STD” group). Additionally, there is a “sexual activity index” describing the frequency of sexual activity by age, peaking at age 22 for females and at age 27 for males, and a matching function that determines the age distribution of sexual partners and their distribution across risk groups, conditional on own age.

**Introducing male circumcision** into the ASSA2008 model requires four adjustments:

- First, for the male population, it is necessary to distinguish circumcised and noncircumcised males, across age and risk groups. This has been accomplished by adding vectors and matrices to the model tracking the circumcised population, by risk group and age, and over time.
- Second, setting a default rule for the *incidence* of male circumcision that endogenously replicates the observed *prevalence* of male circumcision. Department of Health, Medical Research Council, and ORC Macro [2] report a male circumcision rate of 44.7 percent for ages 15–59. This rate increases from 25.5 percent at age 15–19, to 42.9 percent at ages 20–29, to 53.1 percent at ages 30–59. To replicate these numbers, it is assumed that 25.5 percent of the population is circumcised before they reach adulthood, that 23.5 percent of those not yet circumcised get circumcised at age 20, and that 15.3 percent of those not yet circumcised get circumcised at age 30.
- Third, the annual risk of contracting HIV needed to be modeled separately for circumcised and noncircumcised males. This needed some adjustments in the sheets describing the behavior of female sexual partners, adding columns to determine the HIV infection probabilities for circumcised males separately. The modeling implicitly assumes that male circumcision does not affect male sexual risk behavior, broadly in line with current evidence [3], [4], and that the uptake of male circumcision is not correlated with sexual risk behavior.
- Fourth, the process and parameters determining female-to-male transmission of HIV need to be adapted. In the ASSA2008 model, these transmission rates differ across risk groups, from 0.1 percent per sexual intercourse when both partners are from the “RSK” group to 0.5 percent when both partners are from the “PRO” or “STD” groups. These parameters needed to be adapted to account for different risks for circumcised and noncircumcised males. For circumcised males, the analysis followed current practice and available evidence (as summarized, e.g., by WHO and UNAIDS [5]) by assuming that male circumcision reduces the risk of male acquisition of HIV by 60 percent. For noncircumcised males, the female-to-male transition parameters were set so that the model (running from 1980) replicates HIV incidence for the respective risk groups. Consequently, the transmission risks for noncircumcised males were set about 50 percent higher than the corresponding parameters in the ASSA2008 model (and the risk for circumcised males 40 percent lower—i.e., 60 percent lower than the value for noncircumcised males). With these parameters, the adjusted model replicated closely the estimates and projections of the ASSA2008 model (not only for HIV incidence) without these modifications.

**References**

[1] ASSA. ASSA2008 AIDS and Demographic Models–User Guide (beta version). Cape Town: ASSA; 2011.

[2] Department of Health, Medical Research Council, and ORC Macro. 2007, South Africa demographic and health survey 2003*.* 2007. Pretoria: Department of Health.

[3] Gray R, Kigozi G, Kong X, Ssempiija V, Makumbi F, Watya S, et al. The effectiveness of male circumcision for HIV prevention and effects on risk behaviors in a posttrial follow-up study. AIDS. 2012 Mar 13. 26(5): 609–15. doi: 10.1097/QAD.0b013e3283504a3f.

[4] Westercamp N, Agot K, Jaoko W, Bailey RC. Risk compensation following male circumcision: results from a two-year prospective cohort study of recently circumcised and uncircumcised men in Nyanza Province, Kenya. AIDS Behav. 2014;18(9):1764-75. Epub 2014/07/23. doi: 10.1007/s10461-014-0846-4. PubMed PMID: 25047688.

[5] WHO and UNAIDS. New data on male circumcision and HIV prevention: policy and programme implications. WHO/UNAIDS Technical Consultation, Male Circumcision and HIV Prevention: Research Implications for Policy and Programming. Montreux, 6-8 March 2007. Geneva: WHO and UNAIDS; 2007. Available: http://libdoc.who.int/publications/2007/9789241595988_eng.pdf.
